# The role of the endoscopic grading of gastric intestinal metaplasia in assessing gastric cancer risk: A systematic review and meta-analysis

**DOI:** 10.3389/fonc.2022.1018248

**Published:** 2022-11-08

**Authors:** Shuangshuang Fang, Yuhan Fu, Sijing Du, Lin Wang, Xiangli Qing, Xiaoying Luo, Gengqing Song, Yang Yang, Wei Wei

**Affiliations:** ^1^ Department of Gastroenterology, Beijing Key Laboratory of Functional Gastrointestinal Disorders Diagnosis and Treatment of Traditional Chinese Medicine, Wangjing Hospital, China Academy of Chinese Medical Sciences, Beijing, China; ^2^ Department of Internal Medicine, MetroHealth Medical Center/Case Western Reserve University, Cleveland, OH, United States; ^3^ Department of Clinical Medicine, Chengdu University of Traditional Chinese Medicine, Chengdu, China; ^4^ Department of Gastroenterology and Hepatology, MetroHealth Medical Center/Case Western Reserve University, Cleveland, OH, United States

**Keywords:** endoscopic grading of gastric intestinal metaplasia, EGGIM, intestinal metaplasia, accuracy, Sensitivity

## Abstract

**Background and aim:**

Patients with gastric intestinal metaplasia (IM) are at increased risk of gastric cancer (GC). The endoscopic grading of gastric intestinal metaplasia (EGGIM) with high-definition endoscopes has shown the potential to facilitate GC risk stratification. However, a comprehensive review and meta-analysis of published articles are lacking. We conducted a meta-analysis to access the value of EGGIM in the assessment of histological IM.

**Materials:**

Studies were selected from PubMed, Medline, Embase, and Cochrane (last selection, Jun 2022). We extracted relevant data to calculate the accuracy of EGGIM compared with the operative link of gastric intestinal metaplasia (OLGIM) and to calculate pooled odds ratio (OR) with a 95% confidence interval (CI) assessing GC risk with different grading.

**Results:**

Four diagnostic studies and three case-control clinical trials were included in the analysis, which included 665 patients and 738 patients, respectively. Compared with OLGIM III/IV, EGGIM(5-10) had a pooled sensitivity and specificity of 0.92(95%CI 0.86-0.96) and 0.90(95%CI 0.88-0.93), and the area under the curve(AUC) was 0.9702. In assessing early GC, the pooled OR of patients with EGGIM(5-10) was 7.46(95%CI 3.41-16.31) compared with that of EGGIM(0-4).

**Conclusions:**

EGGIM is highly consistent with OLGIM, and patients with EGGIM(5-10) are at a higher risk for early GC. Some heterogeneity in the current research suggests that we need to carry out more strict control of confounding factors.

**Systematic Review Registration:**

[https://www.crd.york.ac.uk/PROSPERO/display_record.php?RecordID=248691], (**Prospero registration number:** 248691)

## Introduction

Gastric cancer (GC) is the fifth most commonly diagnosed cancer worldwide and the fourth most common cause of cancer-related death (1). More than 95% of GC are adenocarcinomas (2). According to Lauren’s classification, gastric adenocarcinomas can be classified into diffuse and intestinal types based on histology (3). The intestinal type is the most common type of gastric adenocarcinoma (4), which develops through a multistep process from gastritis, atrophy, intestinal metaplasia (IM), dysplasia to intestinal-type GC (5). Among those steps, IM is widely recognized as a precancerous stomach mucosal lesion, and patients with IM are at increased risk for intestinal-type GC.

Operative Link on Gastric Intestinal Metaplasia (OLGIM) is a grading standard to risk stratify IM, which involves five biopsy specimens: two from the antrum two from the corpus, and one from the angle of the stomach. Patients with OLGIM stage III-IV are at an increased risk of early gastric neoplasia (HR 20.7; 95%CI 5.04 to 85.6) and may develop early gastric neoplasia within two years (6). A meta-analysis that included three case-control studies reported a 3.99-fold odds ratio(95%CI 3.05 to 5.21) for GC of OLGIM stage III-IV (7). Although studies have supported that OLGIM can effectively risk-stratify GC (8–10), there are still some limitations. There are chances that biopsies may miss certain gastric mucosal lesions because they can present multifocally. Furthermore, the histopathology risk stratification tool can bring an extra burden to the healthcare system (11).

In recent years, image-enhanced endoscopy (IEE), including narrow-band imaging (NBI), linked color imaging (LCI), and blue laser imaging (BLI), has improved the performance of endoscopy (12). IEE can present a better characterization of the mucosal pattern, which is more accurate for endoscopists to make diagnoses based on images. Above this foundation, the endoscopic grading of gastric intestinal metaplasia (EGGIM) score system using IEE technology has been proposed to assess the GC risk based on endoscopic visualization of IM, which shows high concordance with OLGIM. EGGIM is performed by endoscopic evaluation of greater and lesser curvature of the antrum, angulus, and greater and lesser curvature of corpus. Each area was scored according to the extent of intestinal metaplasia (0, no intestinal metaplasia; 1, an area less than or equal to 30%; 2, a size greater than 30%), with a total score of 10 (8). Several studies have shown its potential to facilitate GC risk stratification. However, a comprehensive review and meta-analysis of published articles are lacking.

In this study, we conducted a systematic review and meta-analysis to explore the value of the EGGIM system in the assessment of histological IM and the risk classification of early gastric cancer.

## Materials and methods

### Study design

We performed a systematic search for diagnostic studies and case-control studies that evaluate the diagnostic accuracy of the EGGIM system using the OLGIM system or GC incidence as a gold standard. The protocol for this study has previously been registered with PROSPERO (registration no. 248691). The protocol followed the Preferred Reporting Items for Systematic Reviews and Meta-Analyses (PRISMA) (13) guidelines.

### Search strategy

Four scientific databases (PubMed, Medline, Embase, and Cochrane) were searched by two researchers (WL and QXL) independently from their inception through Oct 14, 2021. Eligible articles published after this date were added manually. The last elicitation date was Jun 25, 2022. The references of identified articles were also checked to retrieve potential missed articles. The detailed search strategy is shown in **Supplementary Materials 1**. Variables were combined with “Endoscopic grading of gastric intestinal metaplasia” or “EGGIM”. Titles and abstracts were read to exclude irrelevant records. Articles not excluded were read through by two reviewers. Disagreements were resolved with a third reviewer (DSJ).

### Inclusion/exclusion criteria

The inclusion criteria were as follows: 1) Diagnostic studies whose primary or secondary outcome was the evaluation of the accuracy of EGGIM using OLGIM as a gold standard, or case-control studies using EGGIM to stratify GC risk. 2) True positive (TP), true negative (TN), false positive (FP), false negative (FN), or the number of GC events were included in the paper. 3) The papers were published in English. We excluded study types, including letters, reviews, expert opinions, study protocols, animal studies, preclinical trials, and studies with fewer than 10 cases.

### Data extraction and quality evaluation

Two reviewers (YY and DSJ) read the included articles and extracted data independently. The following variables were collected: the first author, publication year, country, study period, sample size, gender, the experience of endoscopists, endoscopic technology, and details of the measurement data. All collected data were double-checked by a third reviewer (FSS). Two reviewers(FSS and WL) scored the included diagnostic studies independently for risk of basis according to Quality Assessment of Diagnostic Accuracy Studies (QUADAS-2) (14) and scored the included case-control studies independently for risk of basis according to The Newcastle–Ottawa Scale (NOS) (15). Disagreements were resolved with a third reviewer(WW).

### Data synthesis and statistical analysis

Meta-DiSc 1.4 and RevMan 5.4.1 (Cochrane, London, UK) were used to analyze the data. Heterogeneity was assessed with Cochrane’s Q test and Higgins’s I^2^ statistics. A fixed-effects model was used when heterogeneity was absent (I^2^ <50% or p > 0.1) and a random-effects model was used if heterogeneity existed (50% <I^2^ <80% or p < 0.05). When heterogeneity was substantial (I^2^ >80% or p < 0.01), a descriptive analysis was used, and subgroup analyses implement for different image-enhanced technology when the necessary data were available. All P-values were two-tailed, and P values < 0.05 were deemed to be statistically significant.

## Results

**Figure 1:**
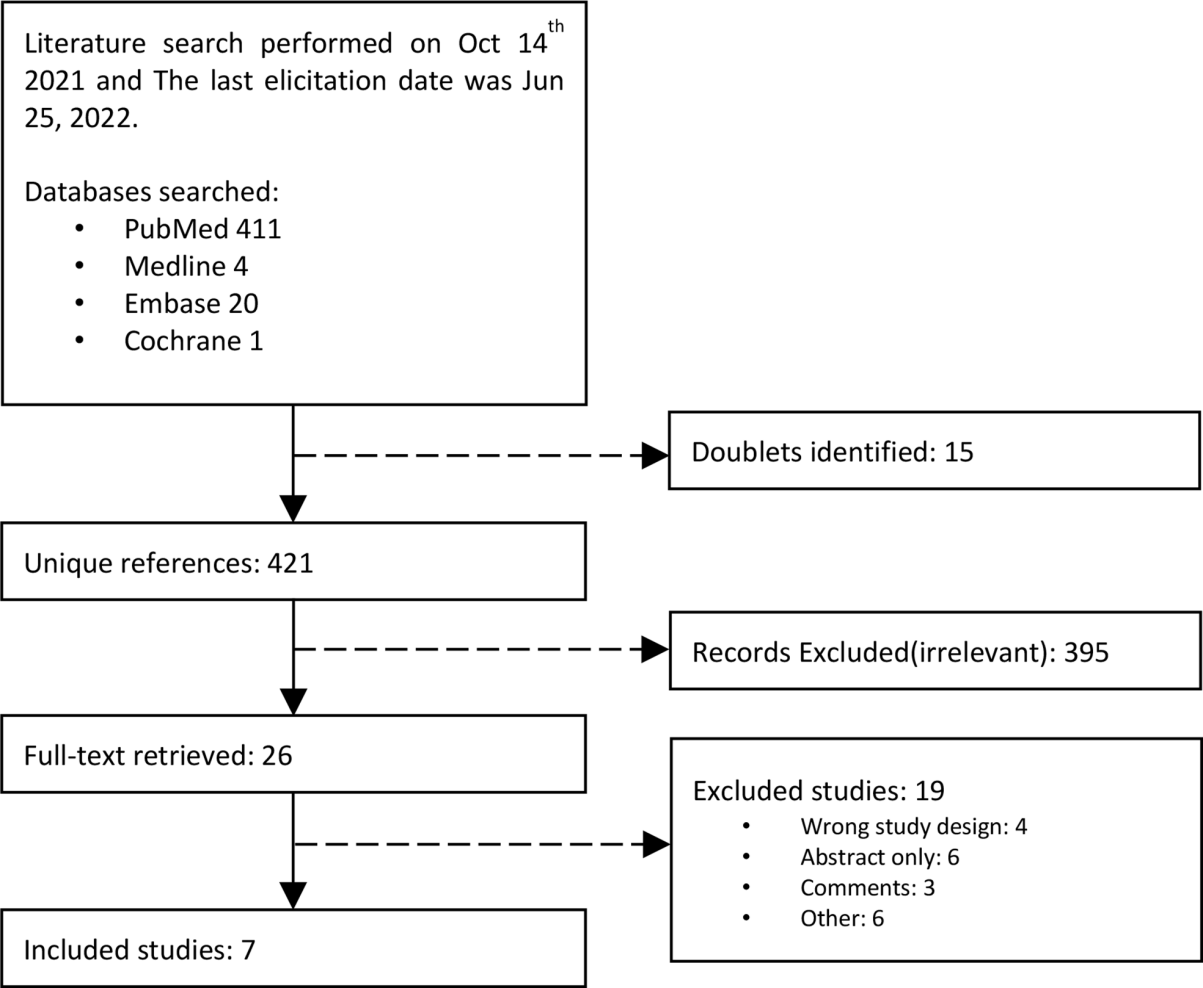
Flow diagram of literature research.

We identified a total of 435 studies. Four diagnostic studies with 665 patients (8, 10, 16, 17) and three case-control studies with 728 patients met the selection criteria (9, 18, 19) (**Figure 1**).

**Table 1** summarizes the characteristics of the studies included in the meta-analysis. Two of the diagnostic studies compared NBI-assisted EGGIM with OLGIM and the other two comparisons were made LCI and BLI respectively. One case-control study compared NBI-assisted EGGIM with OLGIM to classify GC and non-GC patients; one used LCI and one used multiple IEE technologies risks stratify early gastric neoplasia. Most of the trials were conducted in Portugal, of which two were multinational studies and the remaining three were conducted in China and Japan. Four studies stipulate endoscopists’ experience. Two studies required endoscopists who have operated more than 100 NBI per year, one study required endoscopists who performed 200 LCI per year, and the other study required endoscopists who have experience in performing more than 5000 upper endoscopies.

**Table 1:** Characteristics of studies included in the meta-analysis.

First Author	Year	Country	Study Period	Study design	Sample Size	Gender	Characteristics of endoscopists	Endoscopic Technology
Guanpo Zhang	2020	China	May, 2020-July, 2020	Diagnostic study	277	56.32%male	Three endoscopists with LCI experience more than 200 high-resolution endoscopy [HRE]-LCI per year.	LCI
Rui Castro	2019	Portugal	Sep, 2018-Nov, 2019	Diagnostic study	37	Unclear	Unclear	BLI
Gianluca Esposito	2018	Italy, Portugal	Jan, 2016-Sep, 2017	Diagnostic study	250	37.6%male	Four fully trained endoscopists with NBI experience (>100 HR-NBI per year).	NBI
Pedro Pimentel Nunes	2016	Portugal, Italy, Romania, UK, USA	Jan, 2014-Mar, 2015	Diagnostic study	101	42%male	In each center, one or more endoscopists with NBI experience (more than 100 HRE-NBI per year) performed the endoscopy.	NBI
Pedro Marcos	2019	Portugal	2012-2017	Case control	186	55.6%male	Data were collected from patients’ endoscopy reports.	NBI
Masashi Kawamura	2021	Japan	Apr, 2017-Mar, 2019	Case control	380	62.1%male	Endoscopists who had experience of >5000 upper endoscopies in 10 Japanese facilities performed endoscopic procedures.	IEE
Jin Zheng	2022	China	Jan, 2018- Dec, 2021	Case control	162	57%male	Data were collected from patients’ endoscopy reports.	LCI

LCI, linked color imaging; BLI, blue laser imaging; HR-NBI, high-resolution narrow-band imaging; NBI, narrow-band imaging; IEE, image-enhanced endoscopy.

### Quality assessment

The assessment of the risk of bias and applicability concerns for the four diagnostic studies are presented in **Table 2**. The survey conducted by Rui Castro et al. (17) was determined to have a high risk of bias in patient selection and reference standards since they failed to enroll consecutive patients and to include all cases in the analysis. The remaining studies were deemed to have a low risk of bias and applicability concern because the cases were included consecutively, EGGIM was graded before the pathological examination, and the pathologists were blinded when determining the gold standard OLGIM.

**Table 2:** Assessment of risk of bias and applicability concern for all included diagnostic studies.

	Patient selection		Index test (EGGIM)		Reference standard (OLGIM)		Flow & timing
First author	Bias	Applicability	Bias	Applicability	Bias	Applicability	Bias
*Guanpo Zhang*	Low	Low	Low	Low	Low	Low	Low
*Rui Castro*	High	Low	Low	Low	High	Low	Low
*Gianluca Esposito*	Low	Low	Low	Low	Low	Low	Low
*Pedro Pimentel Nunes*	Low	Low	Low	Low	Low	Low	Low

EGGIM, endoscopic grading of gastric intestinal metaplasia; OLGIM, operative link of gastric intestinal metaplasia.

According to the NOS assessment, three case-control studies (9, 18, 19) obtained 6 stars, which represents acceptable study quality shown in **Table 3**.

**Table 3:** Assessment of risk of bias and applicability concern for all included case-control studies.

First author	Is the case definition adequate	Representativeness of the case	Selection of control	Definition of controls	Comparability of cases and controls based on the design or analysis	Ascertainment of exposure	Same method of ascertainment for cases and controls	Non-response rate	Total score
Pedro Marcos	★	★	☆	★	★★	☆	★	☆	6
Masashi Kawamura	★	★	☆	★	★☆	★	★	☆	6
Jin Zheng	★	★	☆	★	★☆	★	★	☆	6

★ means the study in accordance with this entry; ☆ means the study failed to match this entry.

### EGGIM compared to OLGIM

**Figure 2:**
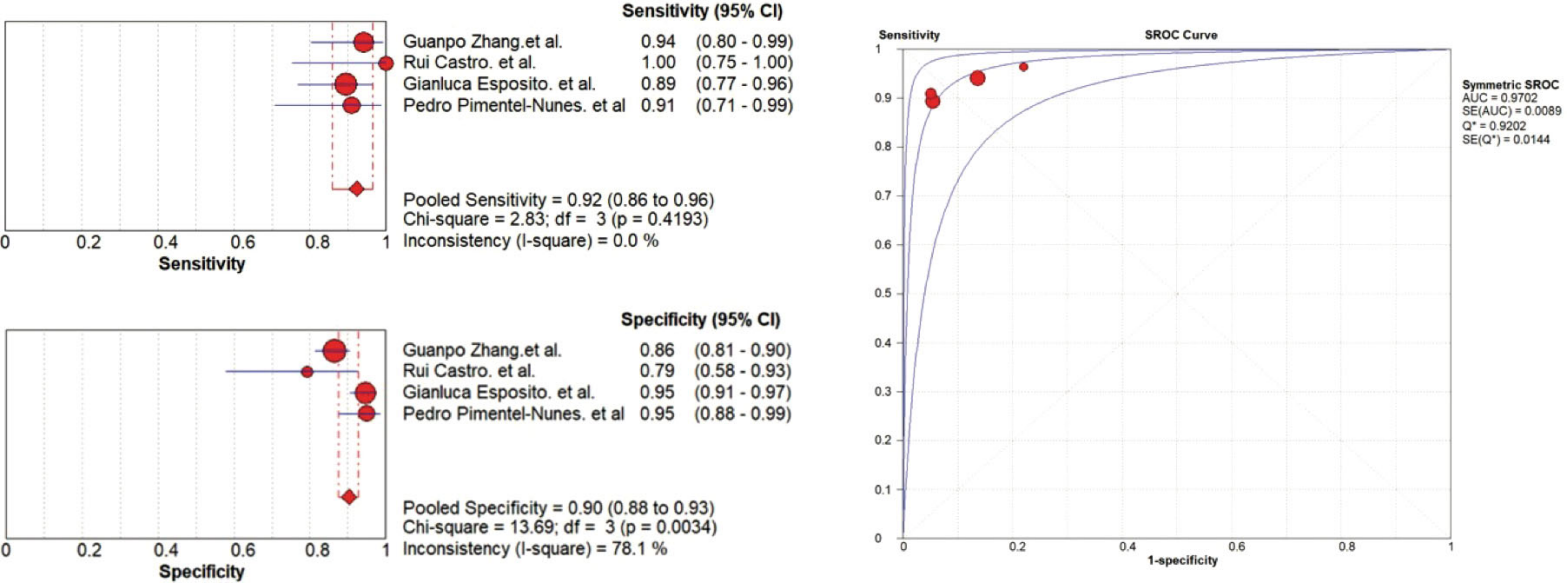
Diagnostic accuracy analysis of EGGIM(5-10) compared to OLGIM III/IV.

Compared with OLGIM III/IV, EGGIM(5-10) obtained a pooled sensitivity and specificity of 0.92(95%CI 0.86-0.96) and 0.90(95%CI 0.88-0.93), and the area under the curve(AUC) was 0.9702 (**Figure 2**).

The results showed heterogeneity in the specificity(*P*=0.0034, *I^2 =^* 78.1%), between the studies, which means there are differences in the diagnosis of true negatives between studies. Rui Castro et al. (13) who compared BLI with OLGIM reported five false positives among 37 patients(13.51%), and Guanpo Zhang et al. (16) who used LCI reported 33 false positives among 277 patients(11.91%). However, two studies (8, 10) that compared NBI with OLGIM reported a lower false positives rate, 11 out of 250 (4.40%) and 4 out of 101 (3.96%) were false positives, respectively.

### EGGIM for gastric cancer risk stratification

**Figure 3:**
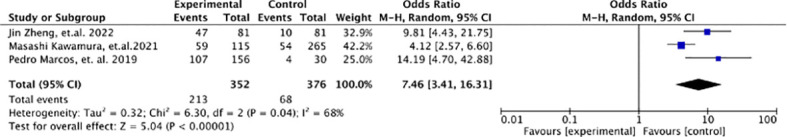
EGGIM(5-10) pooled analysis for early gastric cancer.

A high EGGIM score (5-10) has a significantly higher risk for GC compared with a low EGGIM score (0-4), with an odds ratio of 7.46 (95%CI 2.06-23.05; *P*=0.04, I^2 =^ 68%) (**Figure 3**).

## Discussion

The OLGIM system based on histological examination to assess GC risk has been reported in the Kyoto global consensus (20). However, the clinical utility of OLGIM remains controversial especially in low-risk populations since it requires 5 biopsies. The frequency of OLGIM assessment remains low in daily clinical practice (21). The extent of mucosal lesions positively correlates with GC risk (22), and pathological assessment usually focuses on local areas of the stomach and fails to assess the whole stomach. Furthermore, endoscopic risk stratification tools, such as the Kimura-Takemoto classification, are highly correlated with pathological atrophy, and multi-point biopsies are not required (23). Therefore, endoscopic risk stratification tools play a significant role in gastric cancer risk stratification compared with histology-based risk assessment tools.

Our meta-analysis evaluated the effectiveness of EGGIM as a GC risk stratification tool. EGGIM was compared with the gold standard- the pathology-based OLGIM system. We found that EGGIM was in high concordance with OLGIM with an AUC of 0.9702, and patients with EGGIM (5-10 points) had a 7.46-fold higher risk of developing early GC than those with EGGIM (0-4 points). The EGGIM system as a tool for endoscopic GC risk stratification may be helpful for daily clinical practice.

Meta-analysis of the EGGIM system compared with OLGIM shows heterogeneity in specificity between different studies. The results suggest that there are differences in the number of false-positive cases in the various studies. The EGGIM system overestimated the GC risk for some patients with OLGIM stages 0-II. On the other hand, the EGGIM system has a high sensitivity with no apparent heterogeneity, indicating that EGGIM can identify high-risk patients well. Therefore, EGGIM can identify the high-risk population with an acceptable rate of false positives, which can serve as the first step in risk stratification. An endoscopic-guided biopsy will improve the efficiency of GC screening and reduce the need for pathological examinations.

In addition to EGGIM, Kimura-Takemoto’s endoscopic atrophy classification, which assesses the transition of the fundic-pyloric border (24), is widely used in GC risk stratification. It was consistent with the Operative link for gastritis assessment(OLGA) (25–27). Patients with Kimura-Takemoto endoscopic atrophy classification stages O2-O3 had a higher risk of gastric cancer HR=9.3 (95%CI 1.7-174, *P*=0.007) *(* 28). Kimura-Takemoto’s endoscopic atrophy classification assesses the border of atrophy, and EGGIM assesses the extent of intestinal metaplasia in five gastric sites. Both Kimura-Takemoto classification and EGGIM can predict GC risk, and the combination of the two may be able to improve the accuracy and consistency of GC risk stratification.

The Kyoto classification risk scoring system, which adds observations of intestinal metaplasia, Fhypertrophic fold enlargement, and nodularity to the Kimura-Takemoto system, is also widely used to assess GC risk (29, 30). The study by Masashi Kawamura et al. (19) found that OLGIM stage III/IV, high EGGIM score (5-10), and Kimura-Takemoto open type were risk factors for GC in multivariate analysis; however, the Kyoto classification risk scoring system combined with endoscopic features are not significant(*P*=0.315). The Kyoto classification risk scoring system based on white light endoscopy may result in a lower diagnostic power than IEE. Meanwhile, it incorporates multiple endoscopic features that may fail to provide new risk predictors. Further studies are recommended to explore the clinical utility of these endoscopic features. We hope these endoscopic and pathological risk assessment tools would be complementary to each other and optimally applied in practice.

There are some limitations of this meta-analysis. First, the number of included studies was small, with only 4 diagnostic studies and 3 case-control studies. Second, the specificity of EGGIM compared to OLGIM is heterogeneous, and there is a heterogeneity of EGGIM in predicting GC risk. Due to insufficient literature, subgroup analysis could not be performed for the operating physicians, the endoscopic techniques, etc. The EGGIM stage in one of the included case-control studies (9) was based on the reports, and there may be a significant bias in the evaluation of the EGGIM. Rui Castro’s study (17) did not report the essential characteristics of the included patients and the endoscopist’s experience, which reduced the study’s reliability.

## Conclusion

In summary, our meta-analysis is the first one to synthesize multiple studies to assess the effectiveness of EGGIM. The results showed that EGGIM was a reliable tool for gastric cancer risk stratification. Even though EGGIM carries a false-positive rate, it can still be complementary to pathological biopsy in risk stratification. In addition, with the development of endoscopic technology, endoscopic diagnosis of GC precancerous mucosa has shown great potential, and a unified diagnostic standard may be helpful to promote its application in daily practice. Finally, further prospective studies on endoscopic GC risk stratification systems such as EGGIM are needed to clarify their clinical utility.

## Data availability statement

The original contributions presented in the study are included in the article/**Supplementary Material**. Further inquiries can be directed to the corresponding authors.

## Author contributions

SF: Conceptualization, Methodology, Software, Validation, Formal analysis, Investigation, Data curation, Writing – original draft, Supervision. YF: Investigation, Writing. SD: Validation, Visualization. LW: Investigation, Data curation. XQ: Investigation, Data curation. XL: Investigation, Data curation. GS: Supervision, Project administration,. YY: Supervision, Project administration, Data curation. WW: Conceptualization, Methodology, Supervision, Project administration, Funding acquisition. All authors contributed to the article and approved the submitted version.

## Funding

National Traditional Chinese Medicine Inheritance and Innovation Team Project (No. ZYYCXTD-C-202210). Scientific and technological innovation project of China Academy of Chinese Medical Science (No. CI2021A01008 and CI2021A01820). Science Research Program for TCM Industry (No. 201507001-09).

## Conflict of interest

The authors declare that the research was conducted in the absence of any commercial or financial relationships that could be construed as a potential conflict of interest.

## Publisher’s note

All claims expressed in this article are solely those of the authors and do not necessarily represent those of their affiliated organizations, or those of the publisher, the editors and the reviewers. Any product that may be evaluated in this article, or claim that may be made by its manufacturer, is not guaranteed or endorsed by the publisher.
