# Supplementary material for: The role of the endoscopic grading of gastric intestinal metaplasia in assessing gastric cancer risk: A systematic review and meta-analysis
Source: Front Oncol. 2022 Nov 8;12:1018248. doi: 10.3389/fonc.2022.1018248 (PMC9679375; doi:10.3389/fonc.2022.1018248)

**Supplementary material**

**Search Strategy**

| **Database** | **Number of literatures** |
| --- | --- |
| PubMed | 410 |
| Embase | 20 |
| Cochrane | 1 |
| Medline | 4 |

PubMed:

(endoscopic grading of gastric intestinal metaplasia) OR (EGGIM)


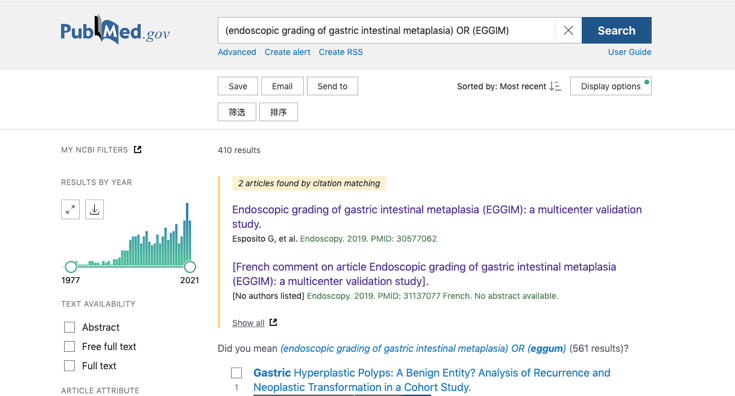


Embase:

'endoscopic grading of gastric intestinal metaplasia':ti,ab,kw OR eggim:ti,ab,kw


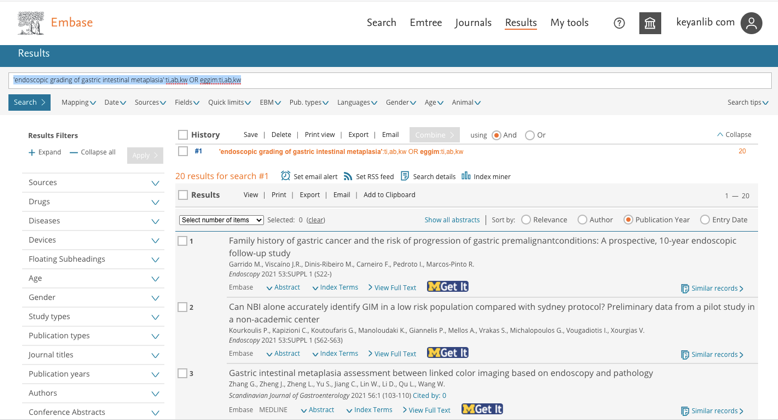


Cochrane

(endoscopic grading of gastric intestinal metaplasia) OR (EGGIM)


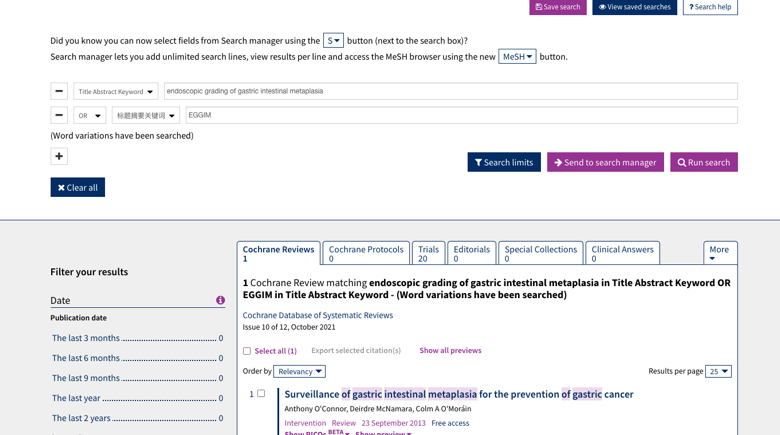


Medline:

(endoscopic grading of gastric intestinal metaplasia) OR (EGGIM)


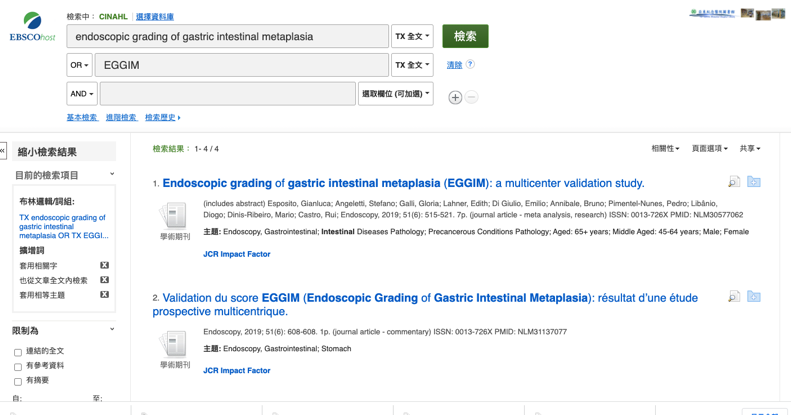

Supplement: Supplementary file 1 [file DataSheet_1.docx]
